# Sequencing data of cell-free DNA fragments in living-related liver transplantation for inborn errors of metabolism

**DOI:** 10.1016/j.dib.2020.105183

**Published:** 2020-01-25

**Authors:** Xiaofan Zhu, Hoi Ioi Ng, Liming Xuan, Yan Long, Yan Mao, Yu Shi, Liying Sun, Bo Liang, Fernando Scaglia, Zhijun Zhu, Kwong Wai Choy

**Affiliations:** a Department of Obstetrics and Gynaecology, The Chinese University of Hong Kong, Hong Kong, China; b Liver Transplantation Center, National Clinical Research Center for Digestive Diseases, Beijing Friendship Hospital, Capital Medical University, Beijing, 100050, China; c Basecare Medical Device Co., Ltd., 218 Xinghu Road, SIP, Suzhou, Jiangsu, 215001, China; d State Key Laboratory of Microbial Metabolism, Joint International Research Laboratory of Metabolic and Developmental Sciences, School of Life Sciences and Biotechnology, Shanghai Jiao Tong University, Shanghai, 200240, China; e Department of Molecular and Human Genetics, Baylor College of Medicine, Houston, TX, USA; f Texas Children's Hospital, Houston, TX, USA; g The Chinese University of Hong Kong-Baylor College of Medicine Joint Center for Medical Genetics, Hong Kong, China

**Keywords:** Graft derived cell-free DNA, Fragment size, Living-related liver transplantation, Inborn errors of metabolism

## Abstract

Graft derived cell-free DNA was recently reported as a non-invasive biomarker to detect graft damage or rejection after liver transplantation. There are a number of methods for quantification of Gcf-DNA,^3^ including quantitative-PCR, digital droplet PCR and massively parallel sequencing (next generation sequencing). Here we present the NGS^4^ data and fragment size distribution of cell-free DNA in the plasma of patients with inborn errors of metabolism who underwent living-related liver transplantation. For more insights please see Analysis of fragment size distribution of cell-free DNA: a potential noninvasive marker to monitor graft damage in living-related liver transplantation for inborn errors of metabolism. [1].

**Table:** Specifications Table

Subject area	*Biology*
More specific subject area	*Genetics and Molecular Biology*
Type of data	*Table, figure*
How data was acquired	*Next generation sequencing (by Ion proton sequencer)*
Data format	*Raw, filtered and analyzed*
Experimental factors	*Cell-free plasma was separated from EDTA blood sample and DNA fragments were extracted by Circulating Nucleic Acid Kit. Library construction was performed by using Ion Plus Fragment Library Kit*
Experimental features	*Sequencing data were aligned to the reference sequences (version: NCBI Build37/hg19) and filtered. Filtered unique reads aligned by TMAP software (version 4.6.11) were used for fragment size analysis and Gcf-DNA quantification.*
Data source location	*China*
Data accessibility	*Raw data are available in China National GeneBank (CNGB) (**https://db.cngb.org/search/project/CNP0000827/**)* *Due to the legal limit of China on management of human genetic resources, the data are controlled by CNGB Data Access (CDA), but interested researchers can submit data permission requeststo CDA and then download and use the controlled data after being approved and authorized by CDA.*
Related research article	*Ng HI etal. Analysis of fragment size distribution of cell-free DNA: a potential noninvasive marker to monitor graft damage in liver transplantation for inborn errors of metabolism. Mol Genet Metab. 2019* [1]

**Table:** 

**Value of the Data** • The data indicate that cell-free DNA in the plasma after liver transplantation is composed of different groups of cell-free DNA fragments. These groups have different size ranges and Graft-derived cell-free DNA is present mainly in the shorter fragments. • The data will be helpful for understanding the physical characteristics of Gcf-DNA^1^ in plasma of patients with inborn errors of metabolism after liver transplantation. • The data presented here will be useful for developing a cost-effective and novel non-invasive method to monitor graft injury after liver transplantation

## 1 Data

Blood specimens from 11 patients with inborn errors of metabolism (IEM) who underwent living-related liver transplantation were collected at six specific time-points (d0, d1, d7, d14, d30, and d60). All the blood samples were drawn in the morning of the given days. This article shows the sequencing data and fragment size profile of cell-free DNA in the plasma of the transplant recipients. The total sequencing reads and reads aligned to Y-chromosome of each patient are summarized in Table 1. Each plasma DNA sample has generated 5.22 ± 1.02 million (mean ± SD) sequencing reads.

**Table 1:** Sequencing reads of the plasma DNA from the 11 inborn errors of metabolism patients underwent liver transplantation.

Case	Sex	Donor	Sex-mismatched	Time-point^a^	Reads aligned to chromosome Y			Reads in all chromosomes		
					Total	105-145bp	>145bp	Total	105∼145bp	>145bp
1	M	Mother	Y	d0	9108	1604	6981	7,153,507	1,165,831	5,205,502
	M	Mother	Y	d1	1160	268	799	3,760,805	1,081,429	2,147,428
	M	Mother	Y	d7	1797	406	1075	4,177,477	998,579	2,618,310
	M	Mother	Y	d14	7105	653	6136	6,102,624	601,390	4,980,364
	M	Mother	Y	d30	7186	798	5991	6,212,544	697,034	4,871,012
	M	Mother	Y	d60	6305	822	5050	5,437,920	683,801	4,112,475
2	F	Father	Y	d0	NA	NA	NA	NA	NA	NA
	F	Father	Y	d1	6763	1894	4375	3,557,290	1,341,454	3,013,608
	F	Father	Y	d7	3884	699	2890	4,866,068	1,173,209	3,078,763
	F	Father	Y	d14	1751	245	1408	3,388,331	765,763	4,128,225
	F	Father	Y	d30	346	49	259	3,813,946	763,552	3,747,196
	F	Father	Y	d60	200	36	138	4,396,055	830,857	4,016,866
3	M	Father	N	d0	5382	790	4209	4,387,036	598,479	3,251,571
	M	Father	N	d1	5651	1508	3705	4,622,284	1,073,034	2,924,354
	M	Father	N	d7	5443	858	4157	4,660,322	668,456	3,348,575
	M	Father	N	d14	4758	644	3925	3,953,748	486,804	3,153,691
	M	Father	N	d30	5918	754	4811	5,030,687	603,692	3,894,321
	M	Father	N	d60	7138	994	5645	6,064,013	802,309	4,442,660
4	F	Mother	N	d0	93	23	56	5,382,863	767,323	3,950,037
	F	Mother	N	d1	101	29	48	5,584,612	1,404,413	3,319,138
	F	Mother	N	d7	72	7	46	4,354,163	920,217	2,787,415
	F	Mother	N	d14	82	10	63	5,599,003	796,692	4,285,613
	F	Mother	N	d30	107	14	69	5,720,462	687,978	4,528,933
	F	Mother	N	d60	86	14	52	4,636,331	565,747	3,675,836
5	F	Father	Y	d0	124	19	89	6,023,962	847,282	4,437,286
	F	Father	Y	d1	5166	1380	3415	6,470,979	1,455,386	4,124,304
	F	Father	Y	d7	2946	488	2235	5,435,357	1,266,813	3,259,309
	F	Father	Y	d14	1866	241	1518	5,819,452	706,961	4,549,664
	F	Father	Y	d30	255	35	187	6,717,742	764,961	5,196,170
	F	Father	Y	d60	281	50	192	6,477,861	789,161	5,026,852
6	M	Mother	Y	d0	4626	556	3752	3,761,858	468,708	2,831,986
	M	Mother	Y	d1	1929	295	1468	4,354,163	920,217	2,787,415
	M	Mother	Y	d7	4413	718	3297	4,824,852	812,866	3,441,641
	M	Mother	Y	d14	5534	621	4668	5,176,431	573,489	4,145,069
	M	Mother	Y	d30	4947	672	4008	4,462,548	568,024	3,464,896
	M	Mother	Y	d60	5878	778	4705	5,295,286	694,273	3,968,044
7	F	Mother	N	d0	62	7	39	3,305,870	464,613	2,418,256
	F	Mother	N	d1	84	16	54	5,644,858	1,012,010	3,832,261
	F	Mother	N	d7	78	10	46	1,637,625	226,575	1,205,254
	F	Mother	N	d14	124	11	97	5,752,959	666,542	4,531,273
	F	Mother	N	d30	71	12	50	5,078,241	610,475	4,008,862
	F	Mother	N	d60	93	15	59	5,625,564	691,607	4,394,909
8	M	Mother	Y	d0	5523	661	4449	4,613,560	547,227	3,498,275
	M	Mother	Y	d1	3796	854	2502	5,565,320	1,435,290	3,226,249
	M	Mother	Y	d7	4986	626	3945	5,322,285	1,025,954	3,620,193
	M	Mother	Y	d14	6149	694	5095	5,550,906	635,790	4,378,132
	M	Mother	Y	d30	7337	796	6161	6,360,163	716,026	4,988,342
	M	Mother	Y	d60	7469	1134	5812	6,533,085	907,020	4,770,280
9	M	Mother	Y	d0	7467	791	6156	5,889,870	643,756	4,515,185
	M	Mother	Y	d1	2990	711	1968	5,435,357	1,266,813	3,259,309
	M	Mother	Y	d7	4894	801	3773	5,999,806	1,005,095	4,411,994
	M	Mother	Y	d14	5705	661	4770	5,122,931	584,550	4,092,357
	M	Mother	Y	d30	6088	688	5073	4,783,192	541,566	3,815,172
	M	Mother	Y	d60	7945	1059	6336	6,923,195	914,019	5,137,872
10	M	Mother	Y	d0	7224	844	5882	5,567,996	684,041	4,196,801
	M	Mother	Y	d1	1438	357	970	4,177,477	998,579	2,618,310
	M	Mother	Y	d7	6117	697	5141	5,492,685	651,822	4,369,007
	M	Mother	Y	d14	6517	749	5433	5,218,482	600,041	4,111,675
	M	Mother	Y	d30	5560	641	4594	4,743,502	587,815	3,654,160
	M	Mother	Y	d60	7571	960	6017	7,094,356	923,938	5,205,815
11	M	Father	N	d0	5840	855	4556	4,714,376	692,469	3,416,575
	M	Father	N	d1	6688	1423	4764	5,322,285	1,025,954	3,620,193
	M	Father	N	d7	7048	1236	5317	5,590,017	925,345	4,071,872
	M	Father	N	d14	7867	758	6721	6,097,298	605,475	4,902,337
	M	Father	N	d30	7554	1051	6080	6,102,619	840,679	4,621,985
	M	Father	N	d60	7763	1002	6253	6,335,392	782,798	4,843,640

a d0 = operation day, sampling before operation; d1 = 1 day after operation; d7 = 7 days after operation; d14 = 14 days after operation; d30 = 30 days after operation; d60 = 60 days after operation.

**Fig. 1:**
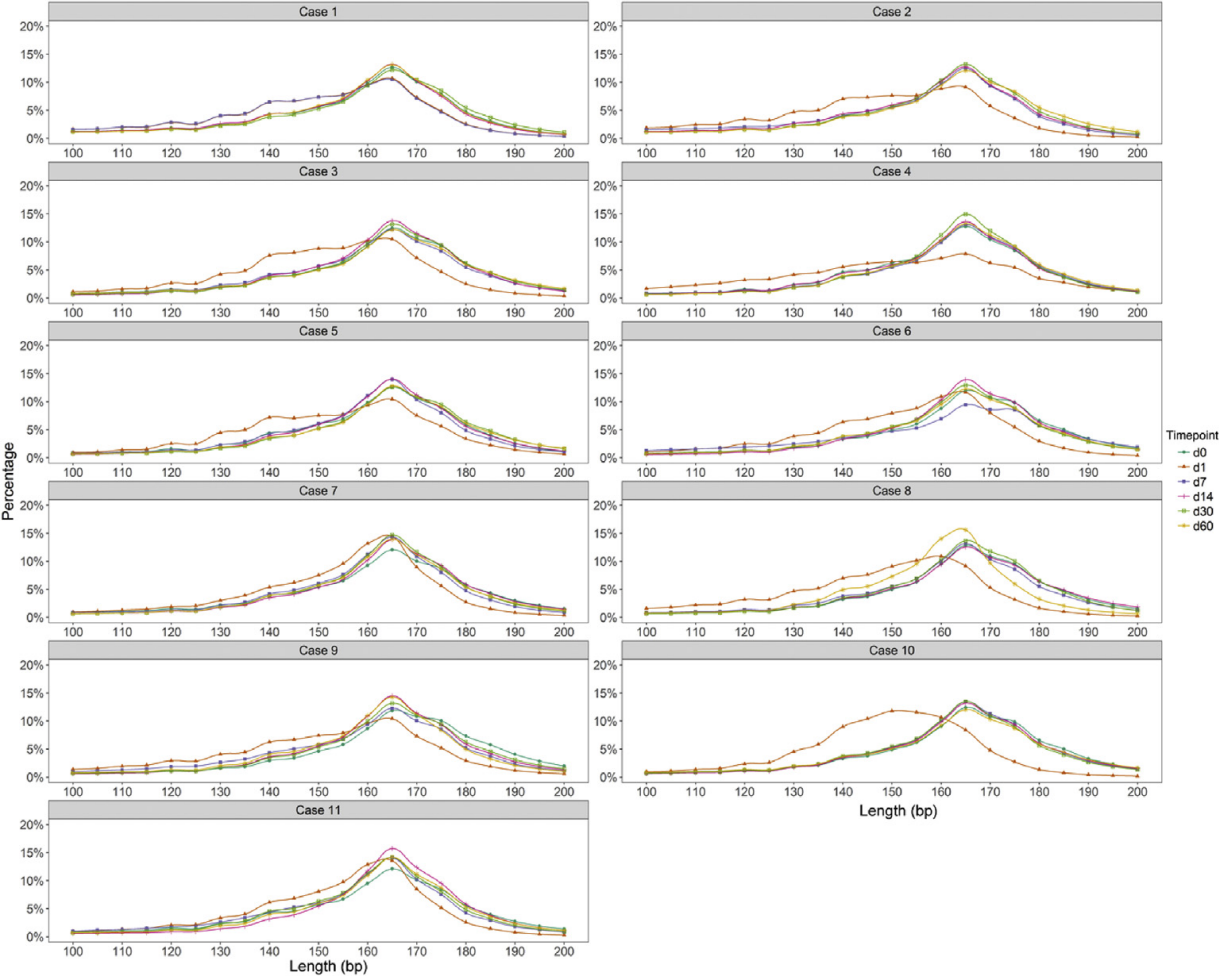
Size distribution of cell-free DNA in the plasma of liver transplantation patients with inborn errors of metabolism (IEM) before and after operation. Each line represents the size distribution of cell-free DNA in the plasma at different dates, with d0 and d1-60 indicating pre-operation and 1–60 days post-operation, respectively.

Analysis of the sequencing read lengths showed that cell-free DNA fragments were routinely present in the circulating plasma with a peak size around 165 base pairs (bp) before operation. Moreover, the size became shorter at post-operative day 1 and returned to a normal size when measured at day 7 or day 14. Despite this finding, there was a noticeable profile difference among participating subjects in the overall size distribution of their cfDNA (Fig. 1). We observed that cell-free DNA fragments in the plasma after liver transplantation were composed of a group with a shorter fragment size (105-145bp) and another group with a longer fragment size (160-170bp) (Fig. 1). An overlap between graft-derived cell-free DNA and recipient-derived cell-free DNA was observed in the intermediate fragment size of 145–160bp. Based on the size distribution of cell-free DNA fragments, the sequencing reads were categorized into two groups (105-145bp, >145bp) (Table 1), which were subsequently used for S/L-Frag calculation [1]. Sequencing reads from Y-chromosome in the sex-matched pairs were used for Gcf-DNA quantification [1].

## 2 Experimental design, materials and methods

### 2.1 Sample preparation and sequencing

Five milliliters of EDTA blood specimens were collected from 11 patients with IEMs, including Ornithine Transcarbamylase Deficiency (OTCD), Propionic Acidemia (PA), Carbamoyl Phosphate Synthetase 1 Deficiency (CPS1D), Primary Hyperoxaluria (PH), N-acetyl Glutamic-acid Synthase Deficiency (NAGSD), Ethylmalonic Encephalopathy (EE) and Methylmalonic Acidemia (MMA), at 6 specific time-points after living-related liver transplantation (i.e. day 0, day 1, day 7, day 14, day 30 and day 60). All of the procedures and informed consent were approved by the Department of Ethics Committee at the Beijing Friendship Hospital of the Capital Medical University (Beijing, China) (approval document number: 2017-P2-080-02). All the legal guardians have provided written informed consent before living donor liver transplantation. Cell-free plasma was separated from the blood samples via two centrifugations (4 °C at 2500×*g* for 10 minutes and 4 °C at 15,500×*g* for 10 minutes). The resultant plasma was stored at −80 °C until further analysis. DNA fragment from 600 μL of cell-free plasma was extracted by using Circulating Nucleic Acid Kit (Qiagen, Germany) [1]. The libraries were constructed by Ion Plus Fragment Library Kit (Life Technologies, USA) on the Ion Proton platform and then quantified using a Qubit Fluorometer. Subsequently, the selected libraries were pooled together with different barcodes and sequenced using an Ion Proton system (Life Technologies).

### 2.2 Sequencing data analysis

All sequencing data were aligned to the human genome reference sequences (version: NCBI Build37/hg19) using TMAP software (version 4.6.11). Unique reads whose mapping quality scores (MAPQs) were greater than 10 and whose lengths were longer than 35 bp were used in subsequent analyses [2]. In the sex-mismatched pairs, the proportion of reads from Y-chromosome (% chrY) was calculated and then used to determine the male DNA concentration in plasma.

### 2.3 Fragment size analysis

The reads mapped to hg19 were converted from Binary Alignment/Map (BAM) format to Browser Extensible Data (BED) format by using BEDTools software, and the length of each read was calculated by subtracting the start of the read from its end in the BED file. The size distribution of cell-free DNA in the recipient plasma was analyzed by calculating the percentage of read counts with each fragment size (ranging from 100 to 200bp) in total read counts. The sequencing reads were then grouped by their lengths, read counts of shorter fragments (105-145bp) and longer fragments (160-170bp) were used for further study on the S/L-Frag calculation in the research paper [1].
